# Health-Seeking Behavior for Noncommunicable Diseases in Rural and Urban Areas of Ernakulam District, Kerala: A Cross-Sectional Study

**DOI:** 10.7759/cureus.104899

**Published:** 2026-03-09

**Authors:** P. Sai Tarun T Reddy, Minali Prathipal, Vaishnavi Vinu, Nihal Noushad, Abhilash Nair, Devika Krishna, Anusree Dinesan, Varsha Prashanth, John Thomson, Rohith Kumar, Devi Unnikrishnan, Brilly M Rose, Akshaya R, Aparna Ajay

**Affiliations:** 1 Community Medicine, Amrita School of Medicine, Amrita Vishwa Vidyapeetham, Kochi, IND

**Keywords:** health-seeking behavior, kerala, non-communicable diseases, primary health care, rural-urban comparison

## Abstract

Background: Health-seeking behavior (HSB) is a crucial determinant of population health and reflects how individuals recognize symptoms and utilize healthcare services. Differences between rural and urban populations influence the management of non-communicable diseases (NCDs), particularly in states like Kerala, where health indicators are high, yet inequities persist.

Objective: To compare patterns and determinants of health-seeking behavior among adults with NCDs in rural and urban areas of Ernakulam district, Kerala.

Methods: A community-based cross-sectional comparative study was conducted in the field practice areas of Amrita Urban Health Centre (Kaloor) and Amrita Community Health Training Centre (Njarackal). Adults aged ≥ 18 years with NCDs such as diabetes, hypertension, thyroid disorders, and cardiovascular diseases were included. Data were collected using a semi-structured questionnaire and analyzed with JAMOVI v2.5.7. Descriptive statistics and chi-square tests were used; p < 0.05 was considered significant.

Results: A total of 168 participants (84 rural, 84 urban) were studied. In rural areas, 54.8% preferred government facilities, whereas 75% of urban residents utilized private or tertiary centers (p < 0.001). Rural residents were 3.63 times more likely to seek care from government hospitals (95% CI 1.89-6.99). Allopathy was the predominant system of treatment (96.4% rural, 95.2% urban). Cost, availability, and accessibility were major determinants influencing the choice of facility. Rural participants depended more on public transport (33.3%) than urban residents (14.3%). Both groups showed high satisfaction levels (≥ 80%).

Conclusion: Urban residents demonstrated more proactive health-seeking behavior, largely due to better access, literacy, and economic resources. Rural populations continue to rely on public healthcare and face constraints related to transportation, affordability, and awareness. Focused interventions are needed to improve accessibility and continuity of NCD care in rural areas.

## Introduction

Health-seeking behavior (HSB) is a critical determinant of the overall health status of a population. It is defined as “any activity undertaken by individuals who perceive themselves to have a health problem or to be ill, for the purpose of finding an appropriate remedy” [1]. An individual’s decision to seek healthcare is influenced by multiple factors, including the type and severity of illness, gender, social environment, cost of care, beliefs regarding disease causation, perceived quality of healthcare services, educational status, and socioeconomic background [2,3]. Health-seeking behavior is inherently complex and dynamic, varying across populations, sociocultural contexts, and time [4].

The healthcare infrastructure of a country plays a pivotal role in shaping health-seeking behavior [2,5]. In developed nations such as the United Kingdom, Canada, Germany, and Japan, universal health coverage (UHC) has ensured relatively equitable access to healthcare services. However, at the global level, only an estimated 56% (95% CI: 44-68) of individuals with non-communicable diseases (NCDs) seek care from formal health facilities. Factors such as low educational attainment, rural residence, and financial constraints have been identified as major determinants of poor health-seeking behavior [6].

In developing countries like India, the attainment of UHC remains a significant challenge due to the predominance of out-of-pocket healthcare expenditure. Concurrently, the burden of NCDs continues to rise, with the number of individuals living with diabetes projected to increase from 40.9 million to 69.9 million by 2025 and obesity expected to affect over 50 million people by 2030 [7,8]. In response, India has launched several initiatives, including the National Programme for Non-Communicable Diseases (NP-NCD) and the India Hypertension Control Initiative (IHCI), aimed at improving early detection, treatment, and long-term management of NCDs [9,10]. Despite these efforts, evidence suggests that nearly three-fourths of individuals with hypertension do not seek appropriate care, resulting in preventable complications and increased morbidity [11]. Furthermore, low health literacy continues to act as a major barrier to timely healthcare utilization, contributing to delayed diagnosis, underuse of available services, and poor adherence to treatment regimens [12].

Kerala, despite its high literacy rates and relatively strong public health system, continues to exhibit disparities in health-seeking behavior between rural and urban populations. Existing evidence indicates that rural residents often depend on public healthcare facilities primarily due to affordability, whereas urban populations increasingly prefer private healthcare providers. A study conducted in rural Gorakhpur reported that although nearly 80% of individuals sought formal healthcare, approximately 60% preferred private institutions, citing perceived quality of care and proximity as key reasons [13]. Similarly, Ratan S. et al. observed that elderly individuals residing in urban areas accessed healthcare services more frequently than their rural counterparts [14]. Other studies from different regions of India have demonstrated that education, gender, and socioeconomic status significantly influence health-seeking behavior for NCDs [15-18]. At the international level, Latunji and Akinyemi [19] reported that higher educational status, income, and health insurance coverage were strongly associated with appropriate healthcare-seeking behavior in Nigeria.

Despite Kerala’s favorable health indicators, there is limited evidence comparing health-seeking behavior among adults with non-communicable diseases (NCDs) in rural and urban settings within the Ernakulam district. A clear understanding of these patterns is essential to identify existing barriers to care and to inform the design of context-specific interventions aimed at promoting equitable healthcare utilization. Therefore, this study aims to compare the patterns of health-seeking behavior related to NCDs among the adult population residing in rural and urban areas of Ernakulam district, Kerala, and to identify the determinants influencing health-seeking behavior for NCDs among these populations.

## Materials and methods

A community-based cross-sectional comparative study was conducted in February 2025 in the field practice areas of the Department of Community Medicine, Amrita School of Medicine. The study was carried out at the Amrita Community Health Training Centre in Njarackal, representing the rural area, and the Amrita Urban Health Centre in Kaloor, representing the urban area, both located in the Ernakulam district, Kerala. These areas are administratively classified as rural and urban regions within the Ernakulam district according to local administrative records.

The study population included adults aged 18 years and above who had been diagnosed with one or more non-communicable diseases commonly managed at the primary care level, including diabetes mellitus, hypertension, thyroid disorders, cardiovascular diseases, or renal diseases. No exclusion criteria were applied in order to capture a broad representation of individuals with non-communicable diseases residing in the study areas.

The sample size was calculated based on findings from a pilot study conducted among adults with non-communicable diseases in areas similar to the study settings within the field practice areas of the Department of Community Medicine, which indicated government-sector healthcare utilization of 90% in rural areas and 60% in urban areas. With a significance level (α) of 0.05 and a power of 90%, the minimum required sample size was estimated to be 42 participants in each group. To enhance the precision of estimates, 84 participants were recruited from each area, resulting in a total sample size of 168. Participants were selected using convenience sampling.

Data were collected using a semi-structured, pre-tested questionnaire that captured information on sociodemographic characteristics, details of diagnosed non-communicable diseases, treatment-seeking preferences, patterns of healthcare utilization, accessibility of healthcare facilities, and levels of satisfaction with the services received. The questionnaire was developed based on a review of relevant literature and was not adapted from any previously validated scale. Following the acquisition of informed consent, data collection was carried out over a two-week period using self-administered Google Forms. Ethical approval for the study was obtained from the Institutional Scientific Research Committee of Amrita Institute of Medical Sciences.

The collected data were analyzed using JAMOVI version 2.5.7. Categorical variables were summarized as frequencies and percentages, while continuous variables were expressed as means and standard deviations. Associations between place of residence (rural or urban) and health-seeking behavior variables were examined using the chi-square test or Fisher’s exact test, as appropriate. Odds ratios with 95% confidence intervals (CI) were calculated, and a p-value of less than 0.05 was considered statistically significant.

## Results

A total of 168 participants were included in the study, with 84 participants each from the rural and urban areas of Ernakulam district.

The mean age of participants from the rural area was 61.9 ± 12.9 years, while that of participants from the urban area was 65.6 ± 10.9 years. Participants aged 60 years and above constituted a higher proportion in the urban area (73.8%) compared to the rural area (64.3%). The gender distribution was comparable between the two groups, with males accounting for 48.8% of participants in the rural area and 42.9% in the urban area. With respect to educational status, the majority of participants in both settings had completed primary or high school education, which represented the largest educational category in the rural (76.2%) and urban (51.2%) populations (Table 1).

**Table 1:** Comparison of Sociodemographic Characteristics Between Rural and Urban Populations (n=168) APL: Above Poverty Line; BPL: Below Poverty Line. Values are expressed as frequency (n) and percentage (%). *Ration card category is used as a proxy indicator of socioeconomic status.

Variables	Category	Rural (N=84) n(%)	Urban (N=84) n(%)
Age (years)	<60	30 (35.7)	22 (26.2)
	≥60	54(64.3)	62 (73.8)
Sex	Male	41 (48.8)	36 (42.9)
	Female	43 (51.2)	48 (57.1)
Education	No formal education	3 (3.6)	1 (1.2)
	Primary to high school	64 (76.2)	43 (51.2)
	Graduate/diploma	13 (15.5)	23 (27.4)
	Professional degree	4 (4.8)	17 (20.2)
Occupation	Professional	7 (8.3)	17 (20.2)
	Skilled	18 (21.4)	7 (8.3)
	Unskilled	13 (15.5)	3 (3.6)
	Unemployed/ homemaker	24 (28.6)	34 (40.5)
	Retired	22 (26.2)	23 (27.4)
Ration card*	APL	49 (58.3)	72 (85.7)
	BPL	35 (41.7)	12 (14.3)
Marital status	Married	73 (86.9)	75 (89.3)
	Unmarried	1 (1.2)	1 (1.2)
	Widow/widower	10 (11.9)	8 (9.5)
Type of family	Joint	10 (11.9)	9 (10.7)
	Nuclear	61 (72.6)	65 (77.4)
	Three generation	13 (15.5)	10 (11.9)

Diabetes mellitus was prevalent in both rural and urban populations, with a marginally higher proportion observed among urban participants (59.5%) compared to those from rural areas (57.1%). Treatment adherence was also slightly higher in the urban group (56%) than in the rural group (51.2%), suggesting comparatively better healthcare access or continuity of care in urban settings. Among participants with diabetes mellitus, a higher proportion of rural residents (26.2%) reported a disease duration of less than five years, whereas urban participants more commonly reported longer disease durations, including 11-20 years (20.2%) and more than 20 years (6%).

Hypertension was more prevalent among urban participants (65.5%) compared to rural participants (59.5%). Similarly, treatment uptake for hypertension was higher in the urban population (61.9%) than in the rural population (50%).

The prevalence of thyroid disorders was comparable between the two groups, accounting for 16.7% of participants in rural areas and 15.5% in urban areas. In contrast, other comorbidities, including renal and cardiovascular diseases, were reported more frequently among rural participants (28.6%) than among urban participants (15.5%). Among individuals with these conditions, treatment uptake was observed in both rural and urban populations, reflecting ongoing healthcare utilization for the management of chronic comorbidities (Table 2).

**Table 2:** Distribution of Health Characteristics Between Rural and Urban Populations DM: Diabetes Mellitus; HTN: Hypertension; CVD: Cardiovascular Diseases. Values are presented as frequency (n) and percentage (%). Duration of DM is calculated among participants with diabetes (Rural n = 49; Urban n = 50). Duration of HTN is calculated among participants receiving treatment for hypertension (Rural n = 42; Urban n = 52). “Others” include renal disorders and cardiovascular diseases.

Variables	Category	Rural n (%) (N=84)	Urban n (%) (N=84)
Diabetes Mellitus	No	35 (41.6)	34 (40.5)
	Yes	49 (58.4)	50 (59.5)
Taking Treatment for Diabetes Mellitus	Yes	43 (87.7)	47 (94.0)
Duration of Diabetes (years) (Rural n=49; Urban n=50)	<5	22 (44.9)	14 (28.0)
	5–10	12 (24.5)	14 (28.0)
	11–20	13 (26.5)	17 (34.0)
	>20	2 (4.1)	5 (10.0)
Hypertension	No	34 (40.5)	29 (34.5)
	Yes	50 (59.5)	55 (65.5)
Taking Treatment for Hypertension	Yes	42 (84.0)	52 (94.5)
Duration of Hypertension (years) (Rural n=50; Urban n=55)	<5	22 (44.0)	20 (36.4)
	5–10	17 (34.0)	17 (30.9)
	11–20	7 (14.0)	13 (23.6)
	>20	4 (8.0)	5 (9.1)
Thyroid Disorders	No	70 (83.3)	71 (84.5)
	Yes	14 (16.7)	13 (15.5)
Other Comorbidities (Renal disorders, CVD)	No	60 (71.4)	71 (84.5)
	Yes	24 (28.6)	13 (15.5)

**Figure 1:**
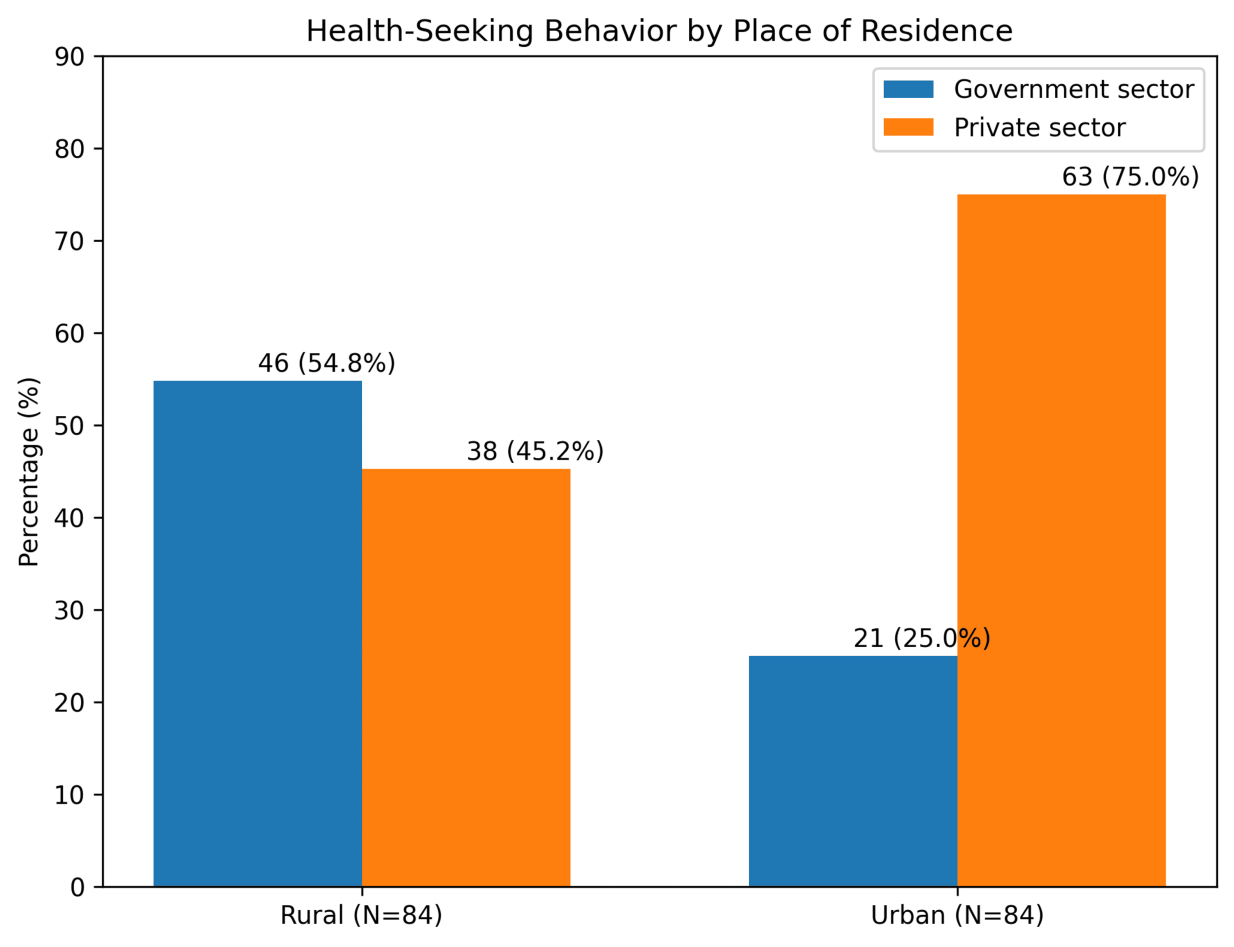
Health-Seeking Behavior by Place of Residence Government sector: Public healthcare facilities; Private sector: Private healthcare facilities. Values are expressed as a percentage (%) of participants seeking care within each place of residence (Rural, n = 84; Urban, n = 84).

A significant difference was observed in the type of healthcare facility utilized. In rural areas, 54.8% preferred government healthcare facilities, while 45.2% sought care from private or tertiary centers. Conversely, in urban areas, 75% of participants preferred private or tertiary care centers, with only 25% utilizing government services (p < 0.001) (Figure 1).

The frequency of consulting a doctor was comparable in both rural and urban settings. Approximately one-third of participants reported seeking medical care monthly, accounting for 35.7% of rural participants and 28.6% of urban participants, while a similar proportion in both groups reported consulting a doctor only when symptoms developed.

A large majority of participants in both settings preferred allopathic systems of treatment, with 96.4% of rural participants and 95.2% of urban participants reporting this preference. Several factors were identified as influencing the choice of healthcare facility, including cost of treatment, availability of healthcare facilities, recommendations from family or friends, perceived severity of symptoms, and accessibility of transportation. Among rural participants, 29.8% reported the cost of treatment as a major determinant, whereas more than half of the urban participants identified the availability of healthcare facilities as the primary influencing factor.

Despite the substantial burden of chronic diseases, most participants reported a low perception of symptom severity. Among those who reported low perceived severity, 49.4% were from rural areas, and 50.6% were from urban areas, while among those who perceived their symptoms as severe, 62.5% were rural participants and 37.5% were urban participants. Accessibility of transportation was reported to be higher among rural participants (32.1%) compared to urban participants (23.8%). Additionally, a small proportion of participants in both groups reported having avoided medical consultation in the past, accounting for 19% of rural participants and 16.7% of urban participants.

Urban participants predominantly reported private hospitals as the nearest healthcare facility (98.8%), whereas rural participants more commonly had government hospitals located nearby (26.2%), a difference that was statistically significant (p < 0.001). With respect to modes of transportation, among participants who used personal vehicles, 42.2% were from rural areas and 57.8% were from urban areas. Among those who walked to healthcare facilities, 46.7% were rural participants and 53.3% were urban participants. In contrast, among those who relied on public transport, 70% were rural participants and 30% were urban participants, and this difference was statistically significant (p = 0.013).

With regard to the distance to healthcare facilities, among participants residing within 1 km, 53.6% were rural residents and 70.2% were urban residents. For distances of 1-5 km, 41.7% were rural, and 28.6% were urban participants, while only a small proportion reported distances greater than 5 km.

There is an association between health-seeking behavior and area of living (p<0.001). People from rural areas are 3.63 (95% CI: 1.89-6.99) times more likely to go to government hospitals for care compared to those living in urban areas (Table 3).

**Table 3:** Association Between Health-Seeking Behavior in Rural and Urban Populations Values are expressed as n (%), with percentages representing row percentages. OR: Odds Ratio; CI: Confidence Interval. Statistical significance was considered at p < 0.05.

Health-Seeking Behavior	Area of Living	Rural n (%)	Urban n (%)	Odds Ratio	95% CI (Lower–Upper)	p-value
Type of Health Facility Utilized	Government sector	46 (68.7)	21 (31.3)	3.63	1.89 – 6.99	<0.001
	Private sector	38 (37.6)	63 (62.4)	Reference	—	

Satisfaction with healthcare services was generally high in both groups. Among those who reported being satisfied, 47.9% were rural participants and 52.1% were urban participants, while among those who were very satisfied, 56.3% were rural and 43.8% were urban participants. No statistically significant association was observed between place of residence and satisfaction with healthcare services (p = 0.491) (Table 4).

**Table 4:** Association Between Determinants of Health-Seeking Behavior in Rural and Urban Populations (n=168) NCDs: Non-Communicable Diseases; AYUSH: Ayurveda, Yoga & Naturopathy, Unani, Siddha, and Homoeopathy. χ²: Chi-square test. †Fisher’s exact test was used where the expected cell frequency was <5. Statistical significance was considered at p < 0.05. Values are expressed as n (%), and percentages represent row percentages, indicating the distribution of responses between rural and urban participants within each category.

Determinants of Health-Seeking Behavior	Category	Rural n (%)	Urban n (%)	χ²	p
Preferred System of Consultation for NCDs	Allopathy	81 (50.3)	80 (49.7)		1.000†
	AYUSH	3 (42.9)	4 (57.1)		
Cost of Treatment	No	59 (46.8)	67 (53.2)	2.03	0.154
	Yes	25 (59.5)	17 (40.5)		
Perceived severity of symptoms	No	79 (49.4)	81 (50.6)		0.720†
	Yes	5 (62.5)	3 (37.5)		
Accessibility of transportation	No	57 (47.1)	64 (52.9)	1.45	0.229
	Yes	27 (57.4)	20 (42.6)		
Does anybody accompany you to the health care facility?	Family member	53 (50.5)	52 (49.5)		0.760†
	Friends	0 (0)	1 (100)		
	Nobody – so doesn't go	3 (75)	1 (25)		
	Self sufficient	28 (49.1)	29 (50.9)		
Nearest health care facility to your home to get NCD Treatment	Government Sector	22 (95.7)	1 (4.3)		<0.001†
	Private Sector	62 (42.7)	83 (57.3)		
Distance to Health care facility	<1 km	45 (43.3)	59 (56.7)		0.060†
	1 to 5 Km	35 (59.3)	24 (40.7)		
	6 to 10 Km	2 (66.7)	1 (33.3)		
	>10 km	2 (100)	0 (0)		
Mode of Transport	Personal Vehicle	35 (42.2)	48 (57.8)	8.64	0.013
	Walking	21 (46.7)	24 (53.3)		
	Public Transport	28 (70)	12 (30)		
Satisfaction level	Neutral	7 (63.6)	4 (36.4)		0.491†
	Not satisfied	1 (100)	0 (0)		
	Satisfied	67 (47.9)	73 (52.1)		
	Very satisfied	9 (56.3)	7 (43.8)		

## Discussion

This comparative study examined sociodemographic characteristics, health status, and health-seeking behavior among rural and urban populations in Ernakulam district and identified notable differences in healthcare access, preferences, and utilization patterns between the two settings.

The mean age of participants was marginally higher in urban areas (65.6 ± 10.9 years) compared to rural areas (61.9 ± 12.9 years). In both populations, a substantial proportion of participants were aged 60 years and above, with a slightly higher representation in urban areas. Marked differences were observed in educational attainment between the two groups. Educational attainment differed notably between the two groups. A higher proportion of participants in urban areas had completed education at the high school level, with a diploma or higher, compared to those in rural areas. In addition, a greater percentage of urban participants possessed professional degrees and were engaged in professional occupations (20.2% vs. 8.3%), which may partly reflect differences in educational attainment and socioeconomic status between rural and urban populations.

With regard to the health status of participants, diabetes mellitus and hypertension were highly prevalent in both rural and urban populations. Diabetes mellitus was reported by 58.4% of rural participants and 59.5% of urban participants, while hypertension was reported by 59.5% and 65.5% of rural and urban participants, respectively. A majority of individuals with these conditions reported currently taking treatment; however, treatment uptake was slightly higher among urban participants (94.0% for diabetes and 94.5% for hypertension) compared to rural participants (87.7% and 84.0%, respectively). These findings reflect differences in health-seeking behavior between rural and urban populations and may be influenced by factors such as accessibility to healthcare facilities, awareness regarding chronic disease management, and availability of healthcare services, which tend to be better in urban settings.

A statistically significant association was observed between the type of healthcare facility utilized and the area of residence. Rural participants predominantly accessed government healthcare facilities (54.8%), whereas urban participants showed a strong preference for private hospitals (75%). Rural residents were found to be more likely to seek care from government facilities (OR = 3.63, 95% CI), and this difference was statistically significant (p < 0.001). Similar patterns have been reported by Tejas Shah et al. in a study conducted in Ahmedabad, where 51.1% of rural residents preferred government and trust hospitals compared to 44.1% of urban residents [20]. The higher utilization of private healthcare facilities among urban populations may be explained by better accessibility, perceived quality of care, and shorter waiting times. The perception of superior services in private hospitals continues to influence healthcare utilization patterns in urban settings [20].

Access to transportation emerged as another important determinant influencing health-seeking behavior. Rural participants were more reliant on public transportation to reach healthcare facilities, whereas urban participants predominantly used personal vehicles, a difference that was statistically significant (p = 0.013). In contrast, a study conducted by Sailesh Bhattarai et al. in Nepal reported that nearly 80% of participants had access to healthcare facilities within a 30-minute walking distance, with walking being the most common mode of transportation (71.3%) [21]. These variations reflect differences in geographic, infrastructural, and transport-related factors that continue to pose challenges to healthcare accessibility, particularly in rural settings.

With regard to treatment modality, both rural and urban populations demonstrated a strong preference for allopathic systems of medicine for the management of non-communicable diseases, with no statistically significant difference between the two groups (p = 1.000). This finding is consistent with observations from a study conducted by Ratan S et al. in Kanpur, where 95.6% of participants reported using allopathic medicine, while only a small proportion utilized alternative systems such as Ayurveda or homeopathy [14].

Overall, the findings of this study reinforce the persistence of a rural-urban divide in health-seeking behavior. Urban residents are more likely to access healthcare services promptly and prefer private healthcare facilities, supported by higher levels of education, income, and closer proximity to healthcare services. In contrast, rural residents continue to encounter structural barriers, including limited availability of healthcare facilities, greater dependence on public transportation, and financial constraints, which may delay or hinder timely healthcare utilization.

This study has a few limitations. The cross-sectional design limits the ability to establish causal relationships between factors influencing health-seeking behavior. The use of convenience sampling may restrict the generalizability of the findings beyond the study population. In addition, reliance on self-reported information may have introduced recall bias.

## Conclusions

This study highlights clear rural-urban differences in health-seeking behavior among adults with non-communicable diseases in the Ernakulam district. Urban residents demonstrated more proactive health-seeking behavior, largely due to better access, literacy, and economic resources, a higher preference for private healthcare providers, and better proximity to services, whereas rural residents relied more on government healthcare facilities and public transportation. Despite a high burden of chronic disease in both settings, the low perception of symptom severity across populations underscores the need for improved health awareness and patient education.

Addressing the identified disparities will require strengthening public healthcare infrastructure in rural areas, improving transportation and accessibility, and enhancing community-based health literacy interventions. Tailored strategies focusing on early care-seeking, continuity of treatment, and equitable service delivery are essential to improve health outcomes for individuals living with non-communicable diseases across both rural and urban settings.

## Appendices

Appendix 1

**Table 5:** Questionnaire PHC: Primary Health Centre; CHC: Community Health Centre; CVD: Cardiovascular Disease; NCDs: Non-Communicable Diseases; APL: Above Poverty Line; BPL: Below Poverty Line. “Multiple response” indicates that participants were permitted to select more than one option. Open-ended items allowed respondents to provide narrative responses.

Section	Question	Response Options
Demographic Information	Age	Open-ended
	Gender	Male / Female / Other
	Area of residence	Rural / Urban
	Education level	No formal education / Primary / Middle school / High school / Intermediate-Diploma / Graduate / Professional degree
	Occupation	Unemployed / Unskilled-Semi-skilled / Skilled-Clerical-Shop-Farm / Semi-professional / Professional / Retired
	Ration card category	APL / BPL
	Number of family members in household	Open-ended
	Marital status	Married / Unmarried / Divorcee / Widow-Widower
	Type of family	Nuclear / Joint / Three-generation
Health Status	Do you have any lifestyle diseases (NCDs)?	Yes / No
	If yes, which NCDs?	Diabetes mellitus / Hypertension / Thyroid disorders / CVD / Renal disorders / Respiratory illness (Multiple response)
	Do you have diabetes mellitus?	Yes / No
	Are you taking treatment for diabetes?	Yes / No
	Duration of diabetes (years)	Open-ended
	Do you have hypertension?	Yes / No
	Are you taking treatment for hypertension?	Yes / No
	Duration of hypertension (years)	Open-ended
	Other NCDs (specify duration and treatment status)	Open-ended
Health-Seeking Behaviour	Primary healthcare system consulted	Allopathy / Ayurveda / Homeopathy / Traditional healer / Self-medication
	Preferred health facility	PHC-CHC / District-Taluk Hospital / Government Medical College / Private Hospital-Tertiary care / Medical shop without consultation
	Frequency of healthcare visit	Monthly / Quarterly / Yearly / Only when symptoms arise
	Reasons for self-medication	Not necessary / Not aware of morbidity / Financial constraints / Waiting for recovery / Distance / No caregiver (Multiple response)
	Access to medications	Prescription / Over-the-counter / Traditional remedies / Self-medication (Multiple response)
	Factors influencing healthcare decision	Cost / Availability / Family recommendation / Personal beliefs / Perceived severity / Transport accessibility (Multiple response)
	Have you ever avoided seeking care?	Yes / No
	If yes, reasons for avoidance	Financial constraints / Lack of facilities / Fear / Cultural beliefs / Lack of time (Multiple response)
	Accompanied to healthcare facility?	Nobody / Self-sufficient / Family member / Friends
Access to Healthcare	Nearest health facility for NCD treatment	PHC-CHC / District-Taluk Hospital / Government Medical College / Private clinic / Private hospital
	Distance to nearest facility	<1 km / 1–5 km / 6–10 km / >10 km
	Mode of transport	Personal vehicle / Walking / Public transport (Multiple response)
	Common facility used in locality	Government hospital / Private hospital / Ayurveda hospital / Homeopathy hospital / Private practitioner / Self-medication
	Satisfaction level	Very satisfied / Satisfied / Neutral / Not satisfied
Awareness and Education	Feel adequately informed about managing condition?	Yes / No
	Received formal education or counselling?	Yes / No
	Sources of information	Healthcare providers / Online resources / Family & friends / Support groups (Multiple response)
	Additional comments or suggestions	Open-ended
